# Do SGLT2 Inhibitors Protect the Kidneys? An Alternative Explanation

**DOI:** 10.2174/0118715303355221241021050443

**Published:** 2025-01-08

**Authors:** Jacob Ilany

**Affiliations:** 1 Sheba Medical Center, Institute of Endocrinology, Tel-Hashomer, Israel

**Keywords:** Diabetes mellitus, neuropathy, SGLT2, microalbumin, proteinuria, renal failure

## Abstract

SGLT2 inhibitors are a family of drugs that were developed to treat diabetes mellitus. In randomized controlled trials, SGLT2 inhibitors seem to prevent kidney deterioration in patients with nephropathies, both diabetic and non-diabetic. However, in contrast to biochemical/physiological results (proteinuria and serum creatinine levels) that improve in all studies, the clinical results (all-cause mortality, cardiovascular death, need for dialysis, or renal transplant) do not consistently improve. In this article, the author would like to suggest that SGLT2 inhibitors do not, in fact, prevent the progression of renal diseases but rather alter laboratory results. This study will present a theory that gives an alternative explanation for the findings in the studies that would explain the above discrepancy between biochemical/physiological and clinical results. In general, the author claims that SGLT2 inhibitors change the kinetics of renal creatinine and microalbumin excretion but do not prevent parenchymal adverse changes in kidneys. This causes a dissociation between renal function markers (such as serum creatinine level and urinary protein) and the real kidney function. Thus, the clinical renal prognosis does not improve despite seemingly better laboratory results.

## 1 INTRODUCTION

SGLT2 inhibitors (SGLT2i) are a family of drugs that were developed to treat hyperglycemia in patients with diabetes mellitus (DM) [1-3]. The drugs work by specifically inhibiting the transporter, whose function is to reabsorb the filtered glucose in the renal glomeruli, thus facilitating glucose excretion in the urine (glycosuria) [4]. However, these drugs are not very effective in treating hyperglycemia and lower hemoglobin A1c levels by only 0.4-0.6% on average in controlled studies [1, 5-7]. In light of the obligation of the FDA to carry out cardiovascular outcome trials (CVOT) for all new medications for diabetes, such studies were conducted for these medications as well. The first published study, EMPA-REG, demonstrated a reduction of more than 30% in overall mortality and cardiovascular mortality in patients with DM who were given empagliflozin [7]. Despite the fact that all the controlled CVOT studies following this study, including empagliflozin itself, failed to demonstrate a similar reduction in overall or cardiovascular mortality (Table **1**) [5-20], these drugs became a hit. Since then, thousands of articles have appeared claiming the effectiveness of this family of drugs for dozens of different medical conditions by many different mechanisms. In fact, there are several articles showing the effect of these drugs on almost every metabolic pathway in the cell [21], many of which claim a direct effect by the drugs, even though it is a very specific blocker for a transporter found exclusively in the renal tubules [22].

The effectiveness of these drugs has indeed been demonstrated in heart failure (HF), where prevention of hospitalizations due to HF was shown in controlled studies (but neither lower total nor CV mortality) [2, 16-18, 20]. This effect could possibly be related to the diuretic effects of SGLT2i [23]. The other condition for which SGLT2i demonstrates consistent effectiveness in randomized controlled trials (RCTs) is kidney disease [24]. Initially, studies showed the prevention of the progression of diabetic nephropathy [12], but very quickly moved to demonstrating effectiveness in many other kidney diseases [8, 9, 19]. These controlled studies demonstrated both a reduction in proteinuria and an inhibition of increasing creatinine levels over time. In addition, an apparent improvement in the clinical end-stage conditions of the kidneys was claimed. These findings are consistent and almost identical in all RCTs with SGLT2i in different patient populations. However, if thoroughly analyzed, in contrast to biochemical/physiological results (proteinuria and serum creatinine levels), the “solid” clinical results (all-cause mortality, cardiovascular death, need for dialysis or renal transplant) do not really improve by using these medications in most RCTs (Table **1**). Nevertheless, SGLT2i became an early prominent step in clinical guidelines for diabetes [25], kidney diseases [24], and cardiac conditions [2, 26, 27] based on these studies.

In this article, the author would like to examine the findings regarding the effect of SGLT2i on renal function in the RCTs. Trying to explain the discrepancy between the biochemical/physiological and the clinical results, the author would like to argue that SGLT2i do not, in fact, really prevent the progression of renal diseases but rather merely alter laboratory results. Further, the author will try to present an alternative explanation for the findings in the studies that would explain the above discrepancy.

## 2 THE EFFECT OF SGLT2I ON THE KIDNEYS

In the articles, the effect of SGLT2i on renal function is being examined on three different levels:

1. Their effect on the glomerular filtration rate (GFR). GFR is reflected by the level of creatinine in the blood. The main parameter is the effect of the drugs on slowing the rate of increasing creatinine levels (=decreasing GFR) in patients with kidney disease.
2. Their effect on the excretion of protein in the urine and the progression of the amount of protein excretion. Microalbuminuria and proteinuria are considered surrogate markers of renal damage.
3. The incidence of clinical consequences of end-stage renal failure: need for dialysis, kidney transplant, and renal death.

The author would like to examine each of these levels and propose a theory that will explain the results of the studies in regard to all of them.

## 3 GFR AND CREATININE

GFR is considered the “gold standard” for assessing kidney function [28]. It can be estimated by measuring various substances (natural or unnatural, such as creatinine, inulin, *etc.*) in a 24-hour urine collection sample and a concomitant measurement of blood levels, and using a simple formula to calculate it. However, due to the inconvenience of urine collections, indirect ways to measure GFR are usually used in clinical studies [28]. In routine patient care and research, the serum creatinine level serves to assess renal function. Although the level of serum creatinine is a fairly good measure of kidney function, there are certain limitations to this index (such as dependence on body muscle mass, ethnic and gender differences, *etc.*). Therefore, different formulas and calculations are used to estimate GFR from serum creatinine levels. All of these formulas introduce some corrections to the aforementioned limitations, but the main component in all of them is still the level of serum creatinine [28].

**Fig. (1):**
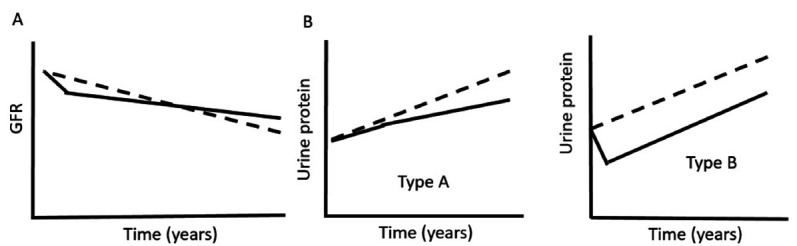
Schematic illustration of the effect of treatment on measured parameters over time. The difference over time between the treatment group (solid line) and the control group (dashed line) for renal parameters is illustrated. (**A**): The change of GFR in the RCTs with SGLT2i. (**B**): Two types of changes in urine protein (see text).

In patients with kidney disease, whether it is diabetic nephropathy or other nephropathy, the level of serum creatinine increases over the years. This reflects a progressive decline in GFR, namely, declining renal function. In all the RCTs carried out with SGLT2i, whether in diabetic patients or those with other kidney diseases, an identical effect of the medications on GFR has been observed [8, 10, 12, 15, 19]. At the beginning of treatment (the first six to twelve months of treatment), the levels of serum creatinine rose above the levels of the control group that did not receive SGLT2i. Namely, the GFR/renal function apparently decreased in these patients compared to the control group at this stage (Fig. **1A**). However, later on, the rate of increasing creatinine was slower in patients on SGLT2i than in those who were not treated. About two years after starting treatment, the GFR in treated patients equaled the level of those who were not treated, and from there, it was higher than in the control groups, *i.e.,* their renal function was apparently better. In light of these results, it was concluded that SGLT2i inhibitors have the property of “renal protection” and are recommended for preventing the progression of a variety of nephropathies [24].

The uniformity of the results in all the RCTs, which were carried out in different populations, different types of kidney diseases, and with different mechanisms of injury, should raise doubts about whether this is indeed a clinical effect on the kidney disease or a biochemical/physiological effect on the measured indices. This uniformity was seen in all the SGLT2i RTCs both in terms of the change in glomerular filtration rate as well as the change in protein excretion in the urine (discussed later). Although theoretical physiological explanations were suggested to explain this phenomenon (such as lowered intraglomerular pressure), we usually do not find such uniformity in clinical studies. Indeed, the “solid” clinical results of these same studies (discussed later) lack such uniformity. All the above points raise the question: Does the effect of SGLT2i on the creatinine level reflect a slowdown in the rate of renal damage, or is it due to a change in the physiology of creatinine and its renal handling?

Serum creatinine levels serve to measure renal function and glomerular filtration rate due to three characteristics [28]:

1. The amount of daily creatinine production in the body is quite constant, correlating with body size.
2. It is freely filtrated in the glomeruli and in direct proportion to its serum level. There is some tubular secretion of creatinine with minimal effect on the ratio between its serum levels and GFR [28].
3. All the filtered creatinine is excreted in the urine since it is usually minimally reabsorbed [29].

These three characteristics are necessary for the serum creatinine level to reflect GFR. Therefore, if even one of these three components is changed, the serum creatinine level will no longer reflect true kidney function. In such cases, even calculating the GFR by urine collection will not necessarily reflect the “true” GFR, *i.e.,* true renal function [28].

Thus, the author proposes that the effect of SGLT2i on serum creatinine levels is caused by two mechanisms. The first phase, in which there is an increase in serum creatinine levels, could be a result (at least partially) of the diuretic effect of these drugs as reflected by the decreased plasma volume caused by said drugs [30, 31]. This diuretic effect and relative dehydration can probably also explain the change in hemoglobin levels in treated patients (discussed later) [32]. On the other hand, the second part of the effect, *i.e.,* the slowing of the rate at which serum creatinine levels increase, could be due to another effect on the aforementioned properties of creatinine clearance. Since there is no reason to believe that SGLT2i inhibitors affect the rate of creatinine production in the body, the author suggests that they increase urinary creatinine secretion. This can theoretically be caused by increasing the active excretion or by decreasing the minimal reabsorption of creatinine. SGLT2i have a similar effect on urinary uric acid secretion. Although other mechanisms caused by SGLT2is affect the serum uric acid levels [33], they increase urine excretion and cause decreased serum uric acid levels [34]. This study hypothesizes that similar effects can occur regarding other metabolites as well. It has been known for many years that creatinine secretion by the kidneys may become very significant under some circumstances, and then it may limit the ability of creatinine to reflect GFR [35]. If we are indeed dealing with such changes in urinary creatinine secretion that affect the serum creatinine level, then the serum creatinine levels no longer reflect the “real” renal function in patients treated with SGLT2i. This is true not only for the first phase of the treatment, where there is no real decline in renal function (but rather relative dehydration), but also for the second phase, where there is no real protection of renal function. The effect of these drugs on creatinine secretion causes a dissociation between the serum creatinine level and the true renal function, and it is no longer a reliable method for measuring renal function. Then, the question arises: How can this theory be tested? It is not simple.

As specified above, the total daily urine creatinine amount is not expected to change as it reflects daily creatinine production. Since the changes in serum creatinine levels are generally slow, even a small change in creatinine secretion can affect the slope of the serum creatinine curve over time. To demonstrate the change by direct measurement of 24-hour urine creatinine, it needs to be corrected for body size, urine volume, and serum creatinine levels, and doing so is not simple and is compounded by the general problems associated with urine collection. Since urine volume is probably slightly higher in SGLT2i-treated patients [31], we can expect their urine creatinine concentration to be lower than that of the control group. This piece of data should actually be available from the RTCs since every measurement of urine microalbumin also measures the urine creatinine concentration. Nevertheless, as explained, this finding will not prove nor disprove the aforementioned theory.

Another way to examine this theory is by using indices other than creatinine to evaluate renal function. One option is by using inulin, but it requires intervention by administering an external substance [28]. Since these are long-term studies with multiple participants, doing so is not practical. It is possible, however, to take a group of patients who are long-term users of the medications and compare the ratio of inulin clearance to creatinine clearance in them and in a control group not treated with these drugs. This way can indeed provide the answer.

### 3.1 Cystatin C

A second option is by measuring cystatin C. Cystatin C is a low molecular weight protein that is increasingly being used to gauge renal function [28]. It is a member of the cystatin superfamily of cystatin protease inhibitors. Cystatin C is freely filtered at the glomerulus and almost completely metabolized in the tubules. Serum cystatin C may be more accurate than serum creatinine in estimating GFR [28].

A recent study compared creatinine- and cystatin C-based GFR estimations in patients with type 2 diabetes with and without SGLT2i treatment [36]. The findings of this study inferred the possibility that SGLT2i may alter the creatinine level while not really affecting kidney function. First, the SGLT2i-treated group had significantly lower creatinine levels than those without SGLT2i, unlike serum cystatin C levels, which were similar between the groups. Second, unlike in patients not treated with SGLT2i, who showed a good correlation between creatinine- and cystatin C-based GFR calculations, in patients treated with SGLT2i, the creatinine-based calculations overestimated the GFR compared to those done by using cystatin C. This fact resulted in more patients in the non-SGLT2i group being classified with higher grades of renal failure. The difference between creatinine- and cystatin C-based GFR calculations (eGFRcr-cys) was significantly higher in the SGLT2i-treated *versus* non-treated group. Although a small study, it implies the possibility that SGLT2i may affect the renal handling of creatinine rather than the true glomerular filtration rate and provides support for the theory presented in this study. This eGFRcr-eGFRcys discrepancy was found by others as well [37].

### 3.2 Microalbumin

The excretion of protein in the urine is a surrogate marker of kidney damage, and it progresses with the progression of kidney damage [38]. This is true in patients with diabetes who develop diabetic nephropathy. Initially, small amounts of albumin are excreted in the urine (microalbuminuria, today called moderately increased albuminuria), and as the renal damage progresses, the amount of protein increases until reaching an amount measured in grams per day (proteinuria). The historical natural course of the appearance of microalbumin in the urine in patients with diabetic nephropathy is as follows: as protein excretion progresses, there is a corresponding decrease in renal function until finally reaching end-stage renal failure and the need for dialysis and a kidney transplant. The introduction of ACE blockers and ARB inhibitors changed this natural course as these drugs delay the progression of proteinuria, sometimes to the point of complete cessation [39]. The above-mentioned phenomenon is not the natural course for all DM patients, as it is well known that some of them develop normoalbuminuric diabetic nephropathy [40]. In these patients, urine microalbumin cannot be used as a surrogate marker for kidney function. Recently, it was shown that “normal range” microalbumin also correlates with complications [41].

SGLT2i also reduces urinary protein excretion in patients with nephropathy (diabetic and non-diabetic) [11, 12, 42]. Treatment with these drugs immediately reduces the amount of protein in the urine, followed by an increase in protein levels at the same rate as those who are not treated with drugs, albeit with a lower curve (since it starts lower) (schematic graph type B as opposed to type A in Fig. **1B**). This kind of graph might be consistent with disease protection when dealing with a causative parameter (like high blood pressure or high LDL cholesterol in regards to vascular disease). However, when dealing with a surrogate marker, such as proteinuria, it does not necessarily indicate a change in the rate of kidney damage but rather an immediate “technical” change that occurs when the drug treatment is initiated and continues throughout the treatment. Such a change can be caused, for example, by increased creatinine excretion in the urine (according to the aforementioned theory). This will result in a lowered microalbumin/creatinine ratio, which is the ratio measured in these studies. It does not necessarily reflect a real effect on renal protein secretion.

Some researchers claim that proteinuria does have a causative role in kidney deterioration, at least in some renal diseases [43]. Nevertheless, SGLT2i show the same “renoprotective” effect in patients without proteinuria or with different degrees of proteinuria [8, 10, 12, 18, 19, 44], and of course, as mentioned above, many DM patients have normoalbuminuric nephropathy [40]. Thus, the change in urine protein amount does not seem to correlate with the effect of SGLT2i on GFR. A similar phenomenon was noticed regarding treatment with ACE inhibitors and AT2 blockers [39]. Furthermore, the immediate improvement in the marker for kidney damage (microalbumin) happens precisely at a time when there is apparently a deterioration in kidney function (increase in blood creatinine level and decrease in GFR) upon starting treatment. This is a consistent phenomenon in all studies and with different starting GFRs (including patients who did not have hyperfiltration to start with), reflecting the dissociation between the two parameters of kidney function (proteinuria and reduced GFR).

Another very important point is that immediately after stopping SGLT2i administration, the proteinuria and GFR return to a placebo-like state [45]. This phenomenon infers an “artificial” effect of the medication rather than a real change in kidney function. If SGLT2i provide real renal protection, why does the effect not last, at least for a while? If treatment with SGLT2i prevents deleterious parenchymal renal changes, then when stopping the treatment, the treated and the control groups should start with different kidney conditions. The deterioration in renal function from that point is expected to be at the same rate, and some differences between the groups should remain.

### 3.3 Hemoglobin

Chronic kidney failure causes anemia. Studies with SGLT2i have shown that they slow the progression of anemia in nephropathy patients [32, 46]. One may claim that this phenomenon is consistent with “renal protection” and the decreased progression of renal damage with SGLT2i. However, the graph of this effect on the hemoglobin level is also of type B (Fig. **1B**) [32, 46]. The difference between the groups occurs immediately after beginning treatment, and the graphs are then parallel. Therefore, this effect also does not indicate renal protection, as the main improvement occurs precisely when the creatinine rises in these patients. Other mechanisms other than improved renal function have been proposed to explain the improved hemoglobin levels, such as enhanced renal and hepatic erythropoietin synthesis caused by SGLT2is [47]. However, as mentioned before, after the first phase of the study, the graphs of the different study groups are parallel, so this mechanism probably cannot be the whole explanation for the difference. Relative dehydration resulting from the diuretic effect of SGLT2i is a possible and more logical explanation for these findings than the improvement of blood production.

### 3.4 “Solid” Clinical Improvement Measures

The aforementioned theory can explain the “biochemical” effects of SGLT2i, but what about the clinical results of the RCTs? Apparently, they consistently show an improvement in the clinical end-points related to kidney disease. But is this true? In this regard, the author would also like to claim that the beneficial clinical effects of SGLT2i have not been proven by the RCTs. In fact, in all studies, the serum creatinine levels were included as an integral part of the clinical outcome measures [5-20]. If, as the author claims, the change in the creatinine level caused by these drugs is a “biochemical/technical” change and does not reflect an improvement in renal function, including creatinine or GFR, then the clinical outcome indices may be misleading. It is reminiscent of the classification of “cardiovascular death or hospitalization for heart failure” as a major clinical outcome after it became clear that there was no improvement in atherosclerotic-related cardiovascular events by using SGLT2i, and only “hospitalization for heart failure” had improved (probably by diuretic effect) [5, 6, 14].

If we take the “real” solid clinical results, *i.e.,* starting chronic dialysis or the need for kidney transplant (and possibly also “renal death”), we will find that in most studies, there was no statistically significant improvement in these results (Table **1**). Furthermore, even with regards to these supposedly “solid” clinical end-points, the effect of serum creatinine level is not eliminated because when making the decision to start dialysis or to refer the patient for renal transplant, the creatinine level is a significant factor that affects the decision. According to the guidelines presented by associations of nephrologists, the creatinine level is included in the considerations for initiating dialysis and the need for renal replacement therapy [48, 49]. Thus, “artificial” change in creatinine levels may lead to different clinical decisions. If the creatinine levels are “biochemically” lower, fewer cases of dialysis and renal transplant are going to be found. In the few studies in which SGLT2i brought about fewer cases of dialysis and renal transplant (Table **1**), each case must be examined individually to see whether the creatinine level influenced the decision to start dialysis or transplantation and whether other clinical indications (acidosis, electrolyte disturbances, *etc.*) were present. Only in doing so can we differentiate a “real” clinical outcome from an “artificial” one.

In most of the large RCTs with SGLT2i, both primarily renal and cardiovascular, no significant reduction in general or cardiovascular mortality was found (Table **1**). This figure reinforces the question of whether these drugs are as clinically beneficial as claimed. The only study that demonstrated a real significant reduction in general and cardiovascular mortality is EMPA-REG [7]. However, there was no significant reduction in mortality in Europe and North America (about 60% of participants) in this study. Similar results were reported by other studies as well [10, 12, 17]. This phenomenon should be investigated and discussed separately. It is puzzling that, although a consistent increase in serum creatinine level was identified in the first six to twelve months in all the studies, none of them were stopped at this point due to safety concerns. On the contrary, the DAPA-CKD study was terminated early due to a decrease in overall mortality [8]. The lower mortality was due to a decrease in infections, malignancy, and heart failure. Despite the attempt of the authors to attribute it to the use of SGLT2i, it did not make sense to stop the study, especially as a decrease in general mortality was not found in other studies with dapagliflozin and almost all the other SGLT2i studies (Table **1**). Furthermore, in the DAPA-CKD study, the more significant difference in overall mortality between the groups was seen in dialysis patients [9], so it is clear that the decrease in overall mortality is not related to renal protection.

Lastly, even if we accept the “solid” renal clinical results in these studies as presented, the Number Needed to Treat (NNT) in order to prevent dialysis and kidney transplantation was high in all these studies (Table **1**). It was between 54 and 81 in the renal studies and much higher in the CV studies. These numbers probably do not justify the use of SGLT2i for this indication.

## CONCLUSION

RCTs with SGLT2i consistently demonstrate a change in the graphs of serum creatinine levels and the amount of protein in the urine. In contrast, they do not show a consistent and clear improvement in real clinical indicators. In this article, the author provides a possible explanation for these changes and why they may not reflect a change in the “real” renal function or “renal protection.” The author claims that SGLT2 inhibitors change the kinetics of renal creatinine and microalbumin excretion but do not prevent parenchymal kidney deterioration. This causes a dissociation between renal function markers (such as serum creatinine level and urinary protein) and the real kidney function. Thus, the clinical renal prognosis does not improve despite seemingly better laboratory results.

The main limitation of this article is that the clinical studies performed to test the SGLT2i effect on the kidney were not intended to discriminate between changes in renal markers and a real reno-protection but rather to rely on the markers and use them to conclude about renal prognosis. Nevertheless, even if it does not prove the hypothesis of this study, an in-depth analysis of the studies and their results provides data that support it. A few steps can be taken to confirm or deny the theory, which are as follows:

1. Conducting studies with other indicators of kidney function (inulin, cystatin C) in patients who are long-treated *versus * those not treated with SGLT2i. This can demonstrate whether there is indeed a dissociation between creatinine level and kidney function in patients treated with SGLT2i.
2. Checking individually in all these studies whether the creatinine level itself influenced the decision to start renal replacement therapy.

If the theory the author has presented is correct, it means that serum creatinine levels do not correctly reflect the true renal function in patients treated with SGLT2i. Moreover, other measures should be used to evaluate renal function in these patients. Furthermore, the fact that SGLT2i-treated patients are less prone to renal replacement therapy without increased mortality, possibly because of “artificially” lower serum creatinine, should raise the question of whether we refer patients for renal replacement therapy too early and whether it can actually be deferred.

Beyond that, even if the clinical result figures do indeed reflect a real improvement in renal function due to the use of SGLT2 inhibitor drugs, the question of whether they should be used for this indication remains open for discussion in light of the high NNT and in light of the side effects, especially ketoacidosis.

## Figures and Tables

**Table 1:** Results of the large RCTs with SLGT2 inhibitors divided by “physiologic/biochemical” and “clinical” results.

Study	Medication	Primary Goal	Type of Pts.	Age of Pts.	No. of Pts.	Length of Follow Up (years)	“Physiologic”/”biochemical” Results		Clinical Results							-	Ref.
							Change of Creatinine Level or GFR		Death From Any Cause	Cardio-Vascular Mortality	Composite Renal Outcome ^a^		Renal Death + Kidney Transplant + Dialysis (no. of Pts.) (%)		P for Dialysis, Transplant or Renal Death	NNT^ b^	
							HR	*P* value			HR	*P* value	Med	Placebo			
EMPA-REG	Empagliflozin	CV	DM	63	7020	2.6	0.56^c^	<0.001	HR – 0.68 *P* <0.001	HR -0.62 *P* <0.001	0.54	<0.001	13 (0.3%)	14 (0.6%)	0.04	333	[7, 15]
EMPA- KIDNEY	Empagliflozin	Renal	Moderate RF + proteinuria	64	6609	2	0.7 ^d^	NAs ^o ^(0.61-0.81)	**NS**	**NS**	0.69	NAs	108 (3.3%)	158 (4.8%)	NAs	66	[19]
EMPEROR- Preserved	Empagliflozin	CV	HF	72	5988	2.2	1.36^e^	<0.001	**NS**	**NS**	0.95	**NS**	20	16	NA	NR	[16, 18]
EMPEROR- Reduced	Empagliflozin	CV	HF	67	3730	1.3	NA	-	**NS**	**NS**	0.5	NAs	6	12	NA	311	[17, 18]
DAPA-CKD	Dapagliflozin	Renal	Mild/moderate RF + proteinuria	62	4304	2.4	0.53^f^	NAs (0.42-0.67)	0.69 (0.53-0.88)^k^	**NS**	0.56	<0.001	73	113	NAs	54	[8, 9]
DECLARE- TIMI 58	Dapagliflozin	CV	DM with CV risk	64	17160	4.2	0.54^g^	<0.001	**NS**	**NS**	0.53	<0.001	11	27	0.012	536	[5, 10]
DELIVER	Dapagliflozin	CV	HF + Mild/ moderate RF	72	6263	2.3	NA	-	**NS**	**NS**	-	-	-	-	**NS ** ^n^	-	[20]
CANVAS	Canagliflozin	CV	DM with CV risk	64	10142	3.5	0.5^h^	NAs (0.3-0.84)	**NS**	**NS**	0.6	NAs	7^l^	14	**NS**	724	[6, 11]
CREDENCE	Canagliflozin	Renal	DM + CKD	63	4401	2.6	0.6^i^	NAs (0.45-0.8)	**NS**	HR – 0.78 *P* = 0.05	0.66	<0.001	78	105	NA	81	[12]
VERTIS-CV	Ertugliflozin	CV	DM + CV	64	8246	3.5	3.1% /3.8%^j^	NA	**NS**	**NS**	0.81	**NS**	7 (0.1%)	3 (0.1%)	**NS**	NR	[14]
SCORED	Sotagliflozin	CV	DM + CKD	69	10584	1.3	NA	-	**NS**	**NS**	0.71	**NS**	?^m^	?	**NS**	NR	[13]

^a^The composite renal outcome is a combined “physiologic/biochemical” and “clinical” parameter as it contains a component of a change of creatinine levels in all the studies.

^b^The NNT to prevent a case of dialysis or renal transplant. The NNT for each study was calculated based on the number of cases in the study.

^c^Doubling of serum creatinine level accompanied by eGFR of ≤45 mL/min/1.73m^2^.

^d^Sustained eGFR decrease ≥40%.; ^e^Estimated GFR mean slope change per year - mL/min/1.73m^2^.

^f^Decline in estimated GFR of ≥50%.; ^g^Sustained eGFR decrease ≥40% to eGFR <60 mL/min/1.73m^2^.

^h^Doubling of serum creatinine level.; ^i^Estimated GFR <15 mL/min/1.73m^2^.

^j^Doubling of serum creatinine level.; ^k^The reason for reduced overall mortality was non-CV death, mainly infections and malignancies, but still, the study was terminated early.

^l^The definition here of end-stage kidney disease also includes a change in creatinine (GFR).

^m^The numbers of Pts are not specified, but even with the change of creatinine level included it is still not significant.

^n^Based on adverse events table.

^o^NAs means that the *P* value was not assessed, but the 95% confidence interval does not include 1.

**Abbreviations:** CKD chronic kidney disease, CV cardiovascular, DM diabetes mellitus, HF heart failure, HR hazard ratio, NA not assessed, NAs not assessed significant, NNT number needed to treat, NR not relevant, NS not significant, RF renal failure.

## LIST OF ABBREVIATIONS

SGLT2i: SGLT2 Inhibitors
DM: Diabetes Mellitus
CVOT: Cardiovascular Outcome Trials
HF: Heart Failure
RCTs: Randomized Controlled Trials
